# Naturally Occurring Variations Modulate the Activity of the HPV33 Early Promoter and its Affinity for the E2 Transcription Factor

**DOI:** 10.1038/s41598-018-33243-y

**Published:** 2018-10-09

**Authors:** Jennifer Alvarez, David Gagnon, François Coutlée, Jacques Archambault

**Affiliations:** 1 Department of Microbiology and Immunology, McGill University, Montreal, QC Canada; 2 Division of Experimental Medicine, McGill University, Montreal, QC Canada; 3 Institut de recherches cliniques de Montréal (IRCM), Montreal, QC Canada; 4 Department of Biochemistry and Molecular Medicine, Université de Montréal, Montreal, QC Canada; 5 Centre de Recherche et Département de Microbiologie Médicale et Infectiologie, Centre Hospitalier de l’Université de Montréal (CHUM), Université de Montréal, Montréal, QC Canada

## Abstract

The human papillomavirus (HPV) Long Control Region (LCR) encompasses the early promoter (EP) that drives transcription of the E6 and E7 oncogenes in keratinocytes and HPV-associated cancers. In this study, the transcriptional activities of the HPV33 EP from the prototype LCR and from eight variants representative of the worldwide diversity of the virus were examined in primary human keratinocytes (PHK) and in the HeLa cervical carcinoma cell line by luciferase reporter-gene assays. Remarkably, the two variations with the greatest effect on the EP in PHK were C7732G and a 79-bp deletion that were associated with high-grade cervical lesions and persistent infections, respectively, in epidemiological studies. In contrast, the three variations most active in HeLa cells were C7537A, A7874C and A7879G. A7874C, which lies within an E2-binding sequence, is also shown to increase the activity and binding of E2 at this site. Collectively, these results indicate that naturally-occurring variations affect the HPV33 EP differentially in PHK than in cancer cells and, furthermore, that they can also alter its regulation by E2. These findings provide a molecular basis for rationalizing the results of previous epidemiological studies and for understanding the contribution of LCR polymorphisms to the oncogenicity and persistence of HPV33 infections.

## Introduction

Infection with high-risk HPVs is associated with cervical cancer and other malignancies of the anogenital tract, as well as with an increasing fraction of head-and-neck cancers, primarily those of the oropharynx^1–3^. HPV33, the subject of this study, is one of the most prevalent types after HPV16 and −18. By itself, HPV33 accounts for 3–5% of all HPV-associated cancers of the female anogenital area^3^, warranting its inclusion in the second generation of nonavalent vaccines (Gardasil®9). Several factors contribute to the progression of HPV-infected cells to cancer, including the genetic and immunological status of the host, the persistence of the infection, a high viral load, integration of the viral DNA into the host chromosomes and, of relevance to this study, variations in the viral genome^4,5^.

HPV types and variants are determined according to the nucleotide (nt) sequence of their L1 open-reading frame (ORF). Viruses whose L1 sequences diverge by more than 10% are considered independent types, while those with less than 10% divergence are defined as variants. The regulatory part of the genome, known as the Long Control Region (LCR), is less evolutionarily constrained than the viral ORFs and can differ by as much as 5% between variants^6^. The LCR is located between the L1 and E6 ORFs and contains the origin of DNA replication which, as its name indicates, is required for replication of the viral genome by the E1 and E2 proteins in conjunction with the host DNA replication machinery. The LCR also encompasses the early promoter (EP) that drives expression of E1 and E2 and of the two oncoproteins E6 and E7, whose primary function is to antagonize the p53 and pRb tumor suppressor pathways, respectively^7,8^. The activity of the EP is dictated primarily by cellular transcription factors such as Sp1, AP1 and NF1 that bind to specific sites in the LCR. E2 is the only viral protein that can regulate the LCR, acting as a transcriptional repressor of the EP when expressed at high levels. This role of E2 is distinct from its function in HPV DNA replication but involves binding to the same sites in the LCR. E2 is frequently not expressed in HPV-associated cancers, often because its ORF has been disrupted upon aberrant integration of the viral genome into the host cell chromosomes. This loss of E2 results in enhanced transcription of E6 and E7 from the EP of the integrated genome, and is believed to be a major mechanism underlying the proliferation of HPV-transformed cells and their resistance to apoptotic death^7,8^.

Naturally-occurring variations in the LCR can potentially alter the oncogenicity of variant viruses by affecting transcription of the early genes. For example, it has been shown that the LCRs of Non-European HPV16 variants are transcriptionally more active than the prototype LCR in reporter gene assays^9^ and that this boost in EP activity increases the ability of the variant genomes to immortalize primary cells and to replicate at higher levels in cell culture models^9–11^. Together, these studies demonstrated that variations in the LCR can modulate the activity of the EP, which in turn can affect the behavior of the virus through changes in early gene expression.

Variations in the LCR have also been linked to different infection parameters and outcomes in epidemiological studies. Our own clinical investigations revealed that HPV33 variants lacking one copy of a tandemly duplicated 79-bp sequence (79del) unique to the HPV33 LCR are associated with persistence of the infection, while those containing a C to G transversion at position 7732 (C7732G) of the LCR are linked to the development of high-grade squamous intraepithelial lesions (HSIL)^12,13^, two risk factors for progression to cervical cancer^14^. However, no information regarding the effect of these nt changes on the activity of the HPV33 EP is available, a gap in knowledge that motivated the current study.

In this manuscript, we used luciferase reporter-gene assays to characterize the EP activity of different HPV33 variants containing 79del and/or C7732G and spanning the worldwide diversity of the virus. We report the identification of specific variations that alter the transcriptional activity of the EP in either primary keratinocytes (PHK) or HeLa cells. We also show that a variation in one of the four E2-binding sites of the HPV33 LCR increases its affinity for this viral regulatory protein. These findings are discussed in light of the unique characteristics of certain HPV33 variants revealed in previous epidemiological studies.

## Results

### Transcriptional activity of the HPV33 prototype EP in PHK and HeLa cells

**Figure 1:**
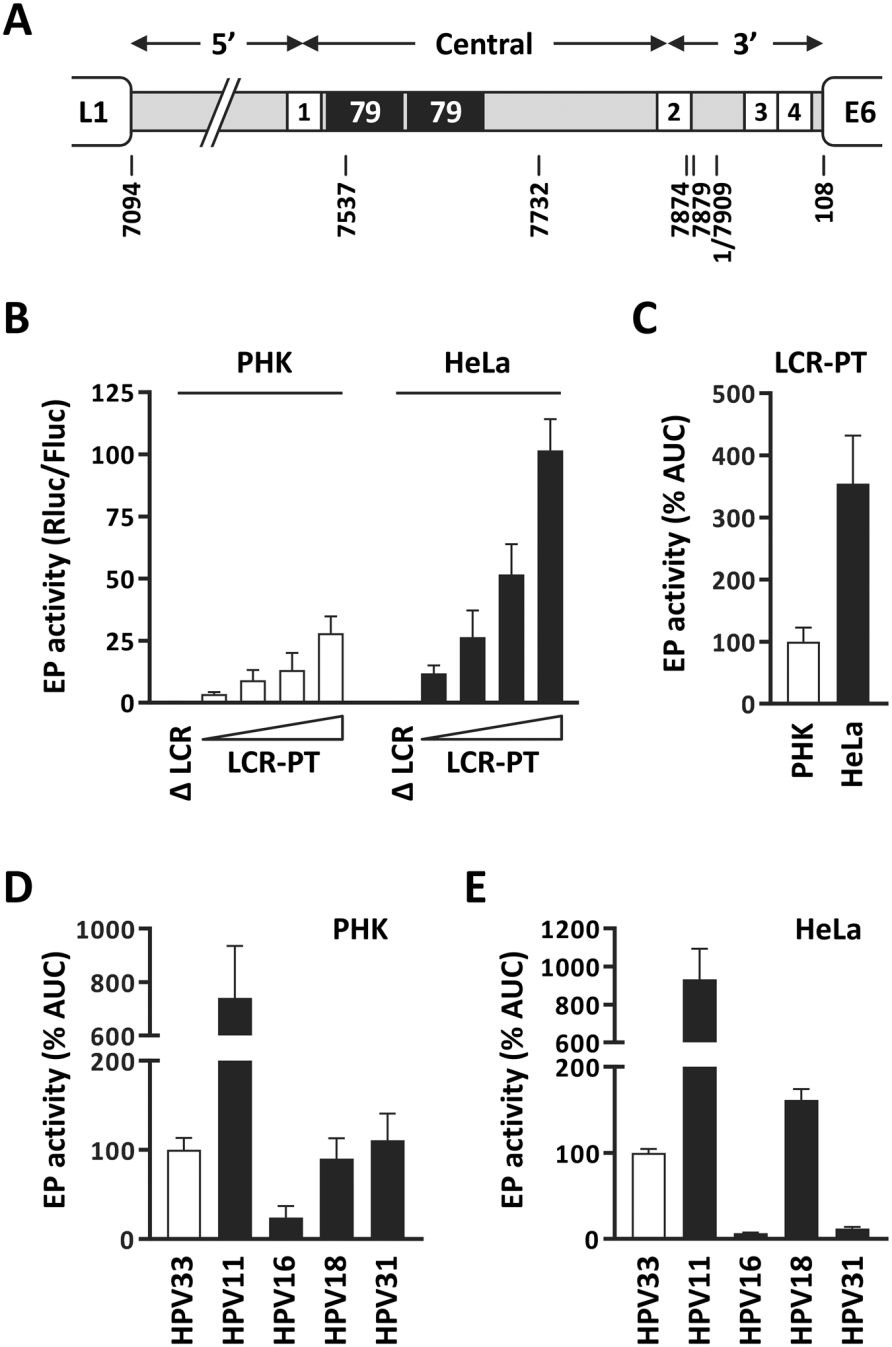
Activity of the HPV33 EP in PHK and HeLa cells. (**A**) Schematic representation of the 924-bp HPV33 LCR (grey line) located between the L1 and E6 ORFs. The locations of the 4 E2-binding sites are indicated by white boxes numbered 1 to 4. Black boxes in the central portion of the LCR represent the duplicated 79-bp region present in the HPV33 prototype. Nucleotide positions indicated below the LCR are numbered according to the HPV33 reference genome (GenBank accession M12732.1). (**B**) EP transcriptional activity of the prototype HPV33 LCR (LCR-PT) in transfected PHK and HeLa cells. The EP activity of LCR-PT was measured in luciferase reporter-gene assays performed with increasing quantities of LCR-PT-Rluc plasmid (25, 50, 100 and 200 ng) to show dose-responsiveness, and a constant amount of LCR-PT-Fluc (25 ng) as an internal control. EP activity is reported as a Rluc/Fluc ratio with each bar corresponding to the mean value derived from a minimum of 4 independent experiments, each performed in duplicate. A Rluc plasmid lacking the LCR was used as a negative control (ΔLCR; 200 ng). Standard deviations (SDs) are indicated by error bars. (**C**) AUC values calculated from the dose-response curves presented in panel B and reported as a percentage of the AUC value (% AUC) obtained in PHK (white bar), which was set 100%. (**D**–**E**) EP activities from the HPV types 33, 11, 16, 18 and 31 LCRs in PHK (**D**) and HeLa cells (**E**).

At the onset of this study, we chose to compare the EP activity of the complete HPV33 LCR (nt 7094-108, Fig. 1A) in undifferentiated PHK, which are characteristic of the host basal epithelial cells infected by HPV, to its activity in the HPV18-positive HeLa cell line as representative of an HPV-transformed cellular environment. Note that we decided to use HeLa cells because an established HPV33-positive cell line is currently lacking and because these cells have been widely used to characterize the EP of different HPV types (for examples see references^15–17^). To measure the activity of the HPV33 EP, a reporter plasmid expressing Rluc under the control of the prototype LCR, herein termed LCR-PT, was constructed. A similar plasmid expressing Fluc from LCR-PT was used as an internal control (note that LCR-PT was chosen as a control to avoid the potential titration of cellular transcription factors that can occur when Fluc is expressed from a strong promoter such as pCMV). The EP activity of LCR-PT was then determined from the Rluc/Fluc ratios of cells transfected with increasing amounts of LCR-PT-Rluc plasmid and a constant amount of LCR-PT-Fluc. The data shown in Fig. 1B revealed that the activity of the HPV33 EP is dose-responsive and substantially higher than that of the negative control plasmid lacking the LCR (ΔLCR). Importantly, it also revealed that the HPV33 EP is less active in PHK than in HeLa cells. To simplify the presentation of these dose-response curves and of similar ones in the remainder of the manuscript, we have used the area under the curve (AUC) as a single integrated measure of EP activity. The AUC values derived from the data presented in Fig. 1B are shown in Fig. 1C, with the AUC determined in PHK set at 100% as the reference. Based on these AUC values, the prototype EP was 3.5-fold stronger in HeLa cells than in PHK, consistent with the overproduction of E6 and E7 observed in HeLa cells and typical of most cervical cancers.

We also compared the EP activity of HPV33 to that of three other high-risk (HPV16, 18 and 31) and one low-risk (HPV11) HPV types that were characterized previously^15–17^. Our results confirmed the stronger, intermediate and weaker EP activity of HPV11, HPV18 and HPV16, respectively, in PHK and HeLa cells^15,16^, as well as the weaker EP activity of HPV31 compared to HPV18 in HeLa cells (Fig. 1D-E)^16,17^. More importantly, the transcriptional activity of the HPV33 EP was found to be robust in both PHK and HeLa cells, and comparable to that of the HPV18 EP (Fig. 1D-E). The finding that the HPV33 EP was as active as the HPV18 EP in HeLa cells provided some reassurance that these cells are an appropriate model in which to study the HPV33 EP, in absence of a well-characterized HPV33-immortalized cell line.

### Sequence and phylogenetic analysis of HPV33 variant LCRs

**Figure 2:**
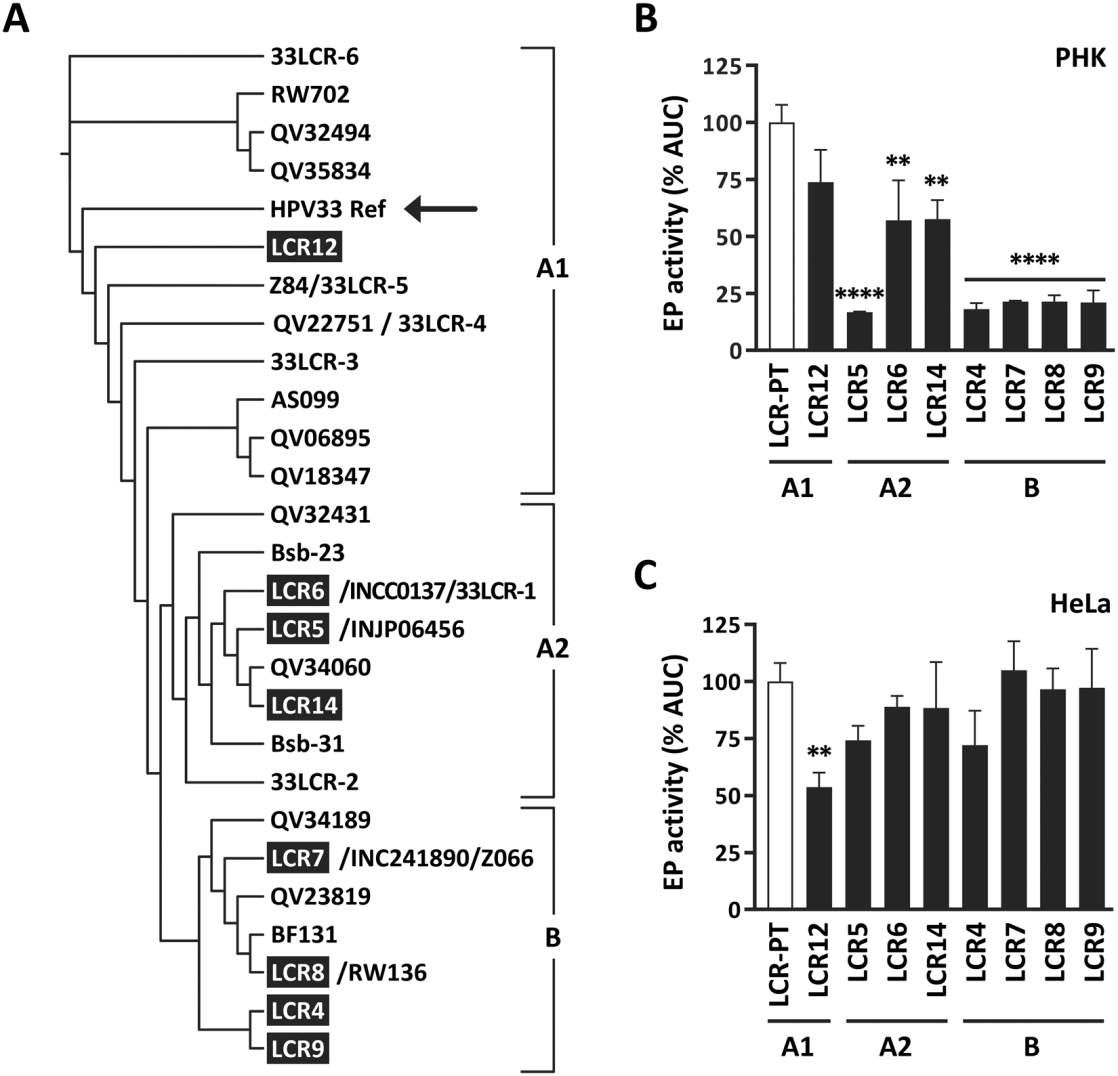
EP activities of LCR variants in PHK and HeLa cells. (**A**) Phylogenetic tree describing the sequence relationships between the 9 HPV33 LCRs characterized in this study and those reported by others. LCRs are grouped into the A1 and A2 sublineages and B lineage, as indicated. The arrow points to the prototype LCR (LCR-PT). Note that several of our variants are identical to those isolated by others from Asian and African cohorts^20,26,27^. LCR5, LCR6, LCR7 and LCR8 corresponds to INJP06456 (Thailand), INCC0137 (Thailand), IN241890 (Thailand) and RW136 (Rwanda), respectively, as indicated in the tree. (**B**,**C**) Bar graphs showing the EP activities of the indicated LCRs in PHK (**B**) and HeLa cells (**C**). EP activities and corresponding SDs are reported as a percentage of the AUC value (% AUC) obtained with LCR-PT (100%, white bar). Each bar represents the mean value obtained from a minimum of 2 independent experiments performed in triplicates in PHK, and of 4 independent experiments performed in duplicates in HeLa cells. Statistical analysis of EP activities was performed using a one-way ANOVA followed by Dunnett’s post-hoc analysis. EP activity values that differ significantly from that of the prototype are indicated by asterisks as follows: *p ≤ 0.05, **p ≤ 0.01, ***p ≤ 0.001, ****p ≤ 0.0001. Variant (sub)lineages are indicated at the bottom of each graph.

Our previous characterization of natural HPV33 LCR variants isolated from cervical samples revealed that a deletion of one copy of the duplicated 79-bp region (79del), was associated with persistent infections, and that the C7732G variation was associated with HSIL in our cohort population^12,13,18,19^. In the current study, we selected eight of these variants for further investigation, focusing on those containing the 79del and/or C7732G variation (LCR 4, 5, 6, 7, 8, 9, 12 and 14 in^12,13^). First, we sequenced the complete LCR (nt 7094-108) of these variants since only a partial sequence (nt 7337–7878) was determined previously. A total of 26 different variations were detected when considering substitutions, insertions and deletions as individual changes (Table 1). LCR8 and LCR9 were the most polymorphic, each carrying 18 variations that together change 1.9% of the LCR sequence compared to the prototype. The phylogenetic tree presented in Fig. 2A shows that the eight variants that we selected for further study belong to the A- and B-lineages^20^ and thus are representative of the worldwide diversity of HPV33 isolates.

**Table 1:** Naturally occurring variations in the HPV33 LCR.

Lineage	Accession #	LCR	5′ region															Central region							3′ region			
		Position	7116	7128	7174	7182	7198	7227	7362	7404	7412	7422	7424	7425	7442	7443	7454	7481	7529	7535	7537 *	7595 *	7686	7732 *	7874 *	7879 *	6	18
		Prototype	T	T	T	G	T	G	T	T	TA	G	A	C	G	C	G	T	-	G	C	79 bp	A	C	A	A	C	G
		Variants	G	C	C	C	C	A	C	A/del	del	T	G	T	A	T	A	G	Ins	A	A	del	C	T/G	C	G	G	A
A1	KT827351	LCR12	—	—	—	—	—	—	—	—	—	—	—	—	—	—	—	—	—	—	—	—	—	T	C	—	—	—
A2	KT827345	LCR5	—	—	—	—	—	A	—	A	—	T	—	—	—	T	A	—	—	A	A	del	—	—	—	G	G	A
A2	KT827346	LCR6	—	—	—	—	—	A	—	A	—	T	—	—	—	T	A	—	—	A	A	del	—	G	—	G	G	A
A2	KT827352	LCR14	—	—	—	—	—	A	—	A	—	T	—	—	—	T	A	—	—	A	A	del	C	G	—	G	G	A
B	KT827344	LCR4	G	C	C	C	C	A	C	del	—	T	G	T	A	—	A	G	—	A	—	del	—	—	—	—	—	A
B	KT827347	LCR7	G	C	C	C	C	A	C	A	—	T	G	T	A	—	A	G	—	A	—	del	—	—	—	—	—	A
B	KT827348	LCR8	G	C	C	C	C	A	C	A	—	T	G	T	A	—	A	G	Ins	A	—	del	—	—	—	—	—	A
B	KT827349	LCR9	G	C	C	C	C	A	C	A	del	T	G	T	A	—	A	G	—	A	—	del	—	—	—	—	—	A

Variant positions of the LCR are numbered according to the sequence of the HPV33 prototype genome (GenBank accession M12732.1). “Ins” refers to the insertion of the sequence CCCTAATA at position 7529 of LCR8. Deletions are indicated by the abbreviation “del”. Hyphens indicate positions that are identical to the prototype. Positions shown in this study to modulate the activity of the EP are indicated by asterisks.

### Transcriptional activities of EP variants in PHK and HeLa cells

The transcriptional activities of the eight variant LCRs and of the prototype were measured in luciferase assays to assess the overall effect of variations on the activity of the HPV33 EP. Several observations were made. First, the two LCRs from the A1-lineage, LCR-PT and LCR12, were the most active in PHK (Fig. 2B). This suggest that the weaker variant LCRs from the A2- and B-lineages carry one or more variations that reduce their EP activity in PHK. This could possibly be the 79del variation that is found in all A2- and B-lineage LCRs but absent from the two A1-lineage LCRs. Second, within the A2-sublineage variants, LCR6 and LCR14 displayed noticeably higher EP activity than LCR5 in PHK (Fig. 2B). This can be attributed to the C7732G variation which is the only difference between LCR6 and LCR5 and is also present in LCR14. Third, since LCR6 and LCR14 displayed virtually identical activities in both PHK and HeLa cells, and only differ by the presence of A7686C in LCR14, we surmise that this variation has little to no effect on EP activity in both cell types. Fourth, the EP activities of the A2- and B-lineage variants were similar to that of the prototype in HeLa cells, revealing that the overall effect of LCR variations is modest in this transformed cell line (Fig. 2C). The only exception was LCR12, which displayed significantly weaker activity than the prototype (p ≤ 0.01, Fig. 2C), suggesting that one or both variations found in this LCR weakens the EP in HeLa cells. Fifth, the B-lineage isolates behaved as a homogenous group, showing comparable activities in both PHK and HeLa cells (Fig. 2C). Given that LCR4, LCR8 and LCR9 differ from LCR7 only by the presence of T7404del, 7529Ins and TA7412del, respectively, this suggests that these variations do not contribute substantially to the activity of the EP in both cell types. The only notable exception was the 30% lower activity of LCR4 compared to LCR7 in HeLa cells (p ≤ 0.01), which could be attributed to the TA7412del variation, the sole difference between both LCRs (Table 1). Collectively, the results presented above show that the EP activities of HPV33 variants differ between (sub)lineages in PHK, but are generally more similar within each group, the lower activity of LCR5 compared to LCR6 and LCR14 being the only noticeable exception. They also highlight that LCR polymorphisms have strikingly different effects in PHK than in HeLa cells.

### The HSIL-associated C7732G variation and 79-bp duplication increase EP activity in PHK

The findings that the HPV33 EP is strengthened by C7732G in PHK (LCR6 vs LCR5 in Fig. 2B) and possibly weakened by 79del in these same cells (A1-sublineage LCRs vs A2- and B-lineage LCRs, Fig. 2B) led us to investigate further the effect of these variations in the context of the prototype EP. Specifically, we introduced C7732G in LCR-PT, either alone or in combination with 79del, as found in the A2-variants LCR6 and LCR14. Similarly, we constructed mutant LCR-PT derivatives in which one or both copies of the 79-bp region were removed by site-directed mutagenesis (79del and 2 × 79del, respectively). Since the single 79-bp region of A2-sublineage LCRs contains the C7537A variation, we also introduced 79del and C7537A together in LCR-PT. Because C7537A lies in the 79-bp region, it has been technically challenging to introduce it into only one of the two identical 79-bp regions of LCR-PT by site directed mutagenesis. Hence, we opted to always study this variation in combination with 79del (79del/C7537A) throughout this study, as both variations are always found together in A1-sublineage variants (Table 1). The activities of these mutant LCRs were then measured in luciferase assays.

**Figure 3:**
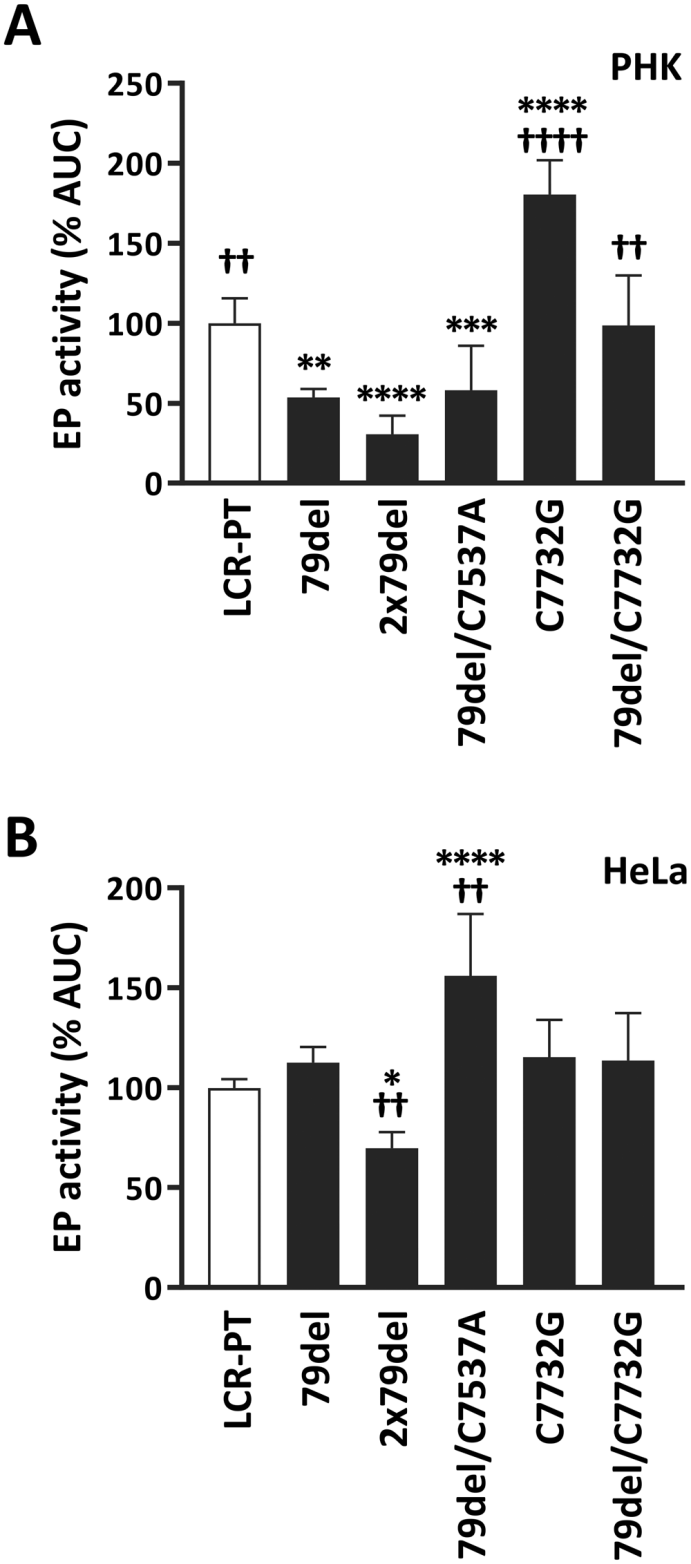
Effect of the 79del and C7732G variations on the transcriptional activity of the prototype EP. (**A**,**B**) Bar graphs showing the EP activities of LCR-PT and mutant derivatives carrying either 79del, 2 × 79del, 79del/C7537A, C7732G or 79del/C7732G, as indicated. EP activities were measured in (**A**) PHK and (**B**) HeLa cells, as described in the legend of Fig. 1, and are reported together with their corresponding SDs as a percentage of the AUC value (% AUC) obtained with LCR-PT (100%, white bar). Statistical significance relative to LCR-PT and LCR-PT 79del is indicated by asterisks (*) and daggers (†), respectively.

The results presented in Fig. 3A showed that C7732G increased the transcriptional activity of LCR-PT by 80%, thus confirming its stimulatory effect in PHK. As expected, C7732G also stimulated transcription from the LCR containing a single 79-bp region (compare 79del to 79del/C7732G in Fig. 3A). In contrast, C7732G had no significant effect in HeLa cells when introduced into LCR-PT, with or without 79del (Fig. 3B), a result consistent with the comparable activities of LCR5 and LCR6 in HeLa cells shown previously (Fig. 2C). Figure 3A also shows that the loss of one of the two 79-bp regions in LCR-PT (79del) reduced EP activity by 50% in PHK, and that removal of both copies (2 × 79del) had an even more severe effect, reducing transcription by 70% (Fig. 3A). The 79-bp duplication appears to be less important in HeLa cells where deletion of one copy (79del) had little to no effect on the EP and removal of both copies (2 × 79del) only reduced its activity by 25% (Fig. 3B). Although the contribution of the 79-bp region was modest in HeLa cells, it could be increased by the C7537A variation which lies within this region (compare 79del to 79del/C7537A in Fig. 3B). A simple interpretation of these findings would be that the 79-bp region is transcriptionally active in PHK, but mostly silent in HeLa cells unless “potentiated” by the C7537A variation.

The results presented above help to explain the transcriptional activities of some variant LCRs. While C7732G certainly accounts for the higher activity of LCR6 and LCR14 in PHK, compared to LCR5, the 79-bp duplication present only in LCR-PT and in LCR12 likely contributes to their higher activities in these primary cells. As for the enhancing role of the 79del/C7537A combination in HeLa cells (Fig. 3B), it was not easily discernable when comparing the activities of A2-sublineage LCRs to those of B-lineage LCRs, which contain and lack this double variation, respectively (Fig. 2C). This raises the possibility that additional polymorphisms in A2-sublineage variants counteract the stimulatory effect of 79del/C7537A in HeLa cells, as examined below.

### The enhancing activity of 79del/C7537A on the EP is antagonized by A7879G in HeLa cells

**Figure 4:**
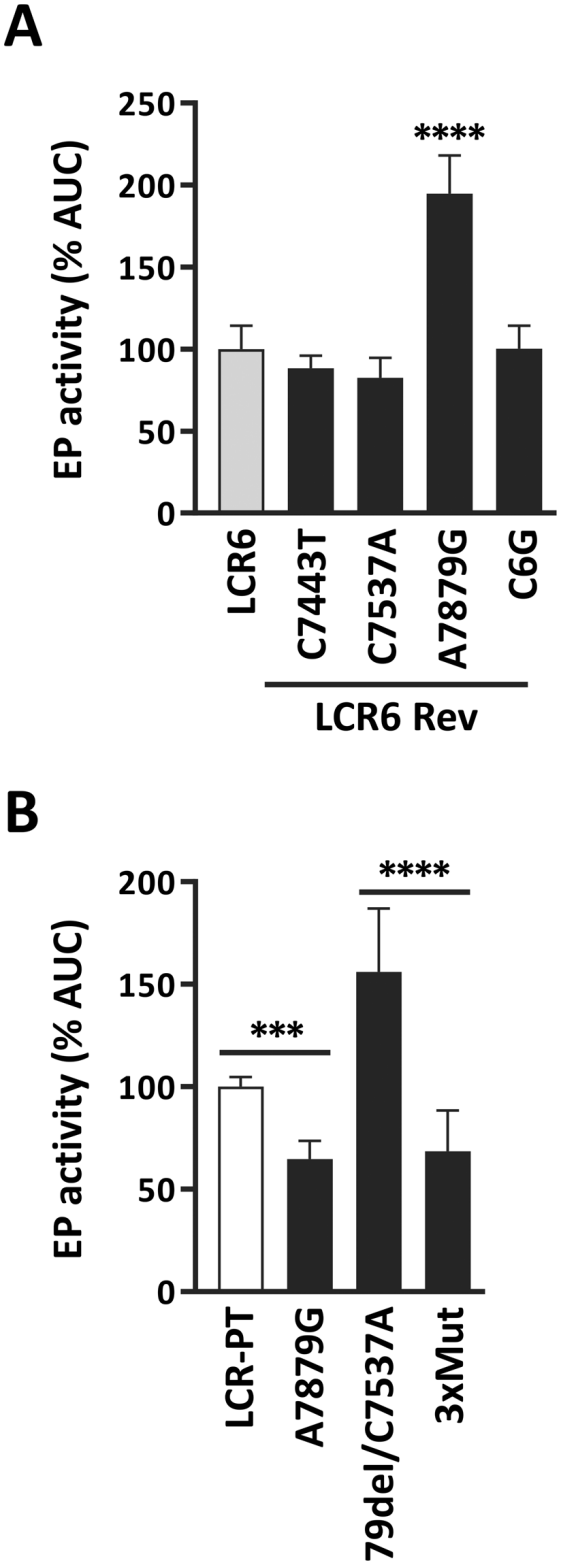
Reversion analysis of the A2-sublineage LCR6 variant in HeLa cells. (**A**) Bar graph showing the EP activities of the indicated LCR6 revertants (LCR6 Rev) in HeLa cells. EP activities were measured as described in Fig. 1 and are reported with their SDs relative to the AUC value (% AUC) of LCR6 (100%, grey bar). Statistical significance relative to LCR6 is indicated by asterisks (*). (**B**) EP activities of LCR-PT and mutant derivatives carrying the indicated variations in HeLa cells. The 3xMut LCR contains the three variations 79del/C7537A/A7879G. EP activities and corresponding SDs are reported relative to the AUC value (% AUC) of LCR-PT (100%, white bar). Statistical significance between pairs of LCRs are indicated by asterisks (*).

The possibility that the stimulatory effect of 79del/C7537A in HeLa cells is masked by other variations in A2-sublineage LCRs prompted us to perform a reversion analysis of LCR6, which contains this double variation. Specifically, we investigated the contribution of the four A2-specific variations, C7443T, C7537A, A7879G and C6G, by reverting them individually back to the sequence of the prototype. Reversion of either C7743T, C7537A or C6G had little to no effect on the strength of the EP (Fig. 4A). In contrast, reversion of A7879G nearly double the activity of the EP, indicating that this variation represses the promoter. These results suggested that A7879G may be the variation that antagonizes the enhancing effect of 79del/C7537A in HeLa cells. To test this possibility directly, A7879G was introduced into LCR-PT either by itself or together with 79del/C7537A; the LCR-PT derivative carrying all three variations (A7879G/79del/C7537A) being termed 3xMut. The results in Fig. 4B showed that A7879G reduced the activity of LCR-PT by 35%, thus confirming that it represses the EP in HeLa cells. Importantly, they also validated the notion that A7879G opposes the stimulatory effect of 79del/C7537A in these cells (compare 79del/C7537A and 3xMut in Fig. 4B). Thus, the presence of A7879G in LCR5, LCR6 and LCR14 LCRs (Table 1) explains why these A2-sublineage LCRs were not more active than the prototype in HeLa cells, even though they contain 79del/C7537A (Fig. 2C). Collectively, these results highlighted the repressive effect of A7879G on the EP and its antagonistic relationship with 79del/C7537A in HeLa cells.

### The two A1-specific variations C7732T and A7874C have opposite effects on EP activity

The A1-sublineage variant LCR12 was the only LCR that showed statistically lower activity than the prototype in HeLa cells (Fig. 2C). LCR12 contains only two variations, C7732T and A7874C, that are unique to the A1 sublineage and are reminiscent of the A2-specific variations C7732G and A7879G investigated above. C7732T affects the same nucleotide as C7732G, changing it for a T rather than a G residue. As for A7874C, it alters a nucleotide that is located only 5-bp away from the one changed by A7879G, thus raising the possibility that both variations affect the same DNA regulatory element. To determine the contribution of C7732T and A7874C to the EP activity of LCR12, we introduced them individually into the prototype and measured the transcriptional activities of the resulting mutant derivatives. Note that since LCR12 contains only 2 variations, introducing C7732T in LCR-PT is equivalent to reverting A7874C in LCR12. Likewise, introduction of A7874C in LCR-PT is identical to reversion of C7732T in LCR12.

**Figure 5:**
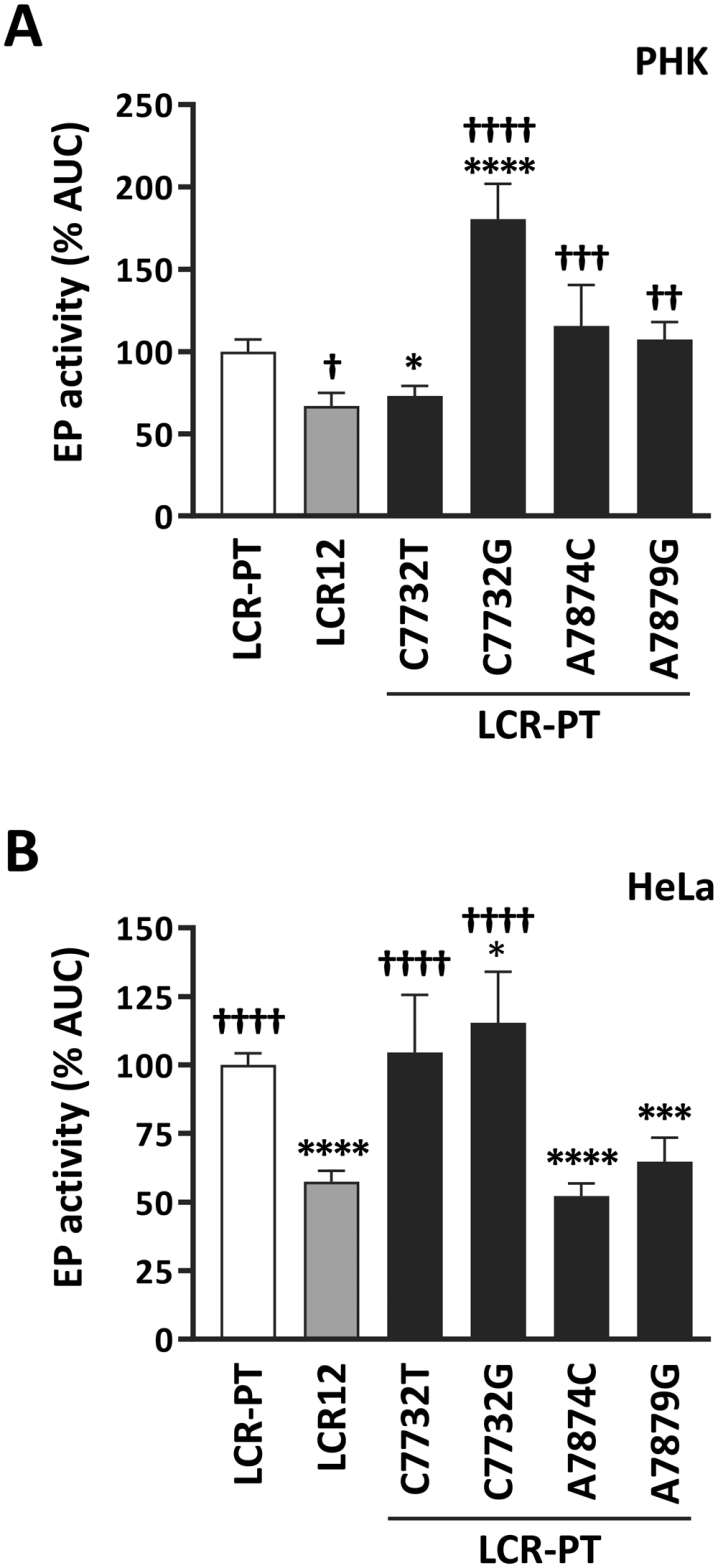
Effect of the two A1-specific variations C7732T and A7874C on the activity of the EP in PHK and HeLa cells. EP activities of LCR12 (grey bar) and of LCR-PT mutant derivatives in (**A**) PHK and (**B**) HeLa cells. The variations introduced in LCR-PT are indicated. EP activities were measured as described above and are reported relative to the AUC value (% AUC) of LCR-PT (100%, white bar). Statistical significance relative to LCR-PT and LCR12 is indicated by asterisks (*) and daggers (†), respectively.

The data in Fig. 5 shows that C7732T decreased the EP activity of LCR-PT by 25% in PHK, down to the level measured for LCR12, while A7874C had little to no effect. Stated otherwise, reversion of C7732T increased the activity of LCR12 by 75% whereas reversion of A7874C had no effect on this LCR. These results indicate that C7732T represses EP-driven transcription in PHK. The situation in HeLa cells was almost opposite. In this cell line, C7732T has little to no effect while A7874C was repressive, reducing EP activity by 50% when introduced in LCR-PT.

Notably, the effects of C7732T on the EP were quite different than those of the C7732G variation, which are shown in Fig. 5 for comparison. While C7732T reduced EP activity in PHK, C7732G activated transcription in these cells, as noted earlier. Furthermore, whereas C7732T stimulated EP activity in HeLa cells, C7732G had no significant effect. These results argue that it is the identity of the variant nucleotide at position 7732, rather than the simple loss of a C residue, that dictates the effects of these two variations.

In summary, the results presented above indicate that C7732T represses the EP in PHK, in contrast to C7732G which activates it. They also show that A7874C represses the EP in HeLa cells, similarly to A7879G.

### Transactivation by the viral E2 protein is enhanced by the A1-specific A7874C variation

**Figure 6:**
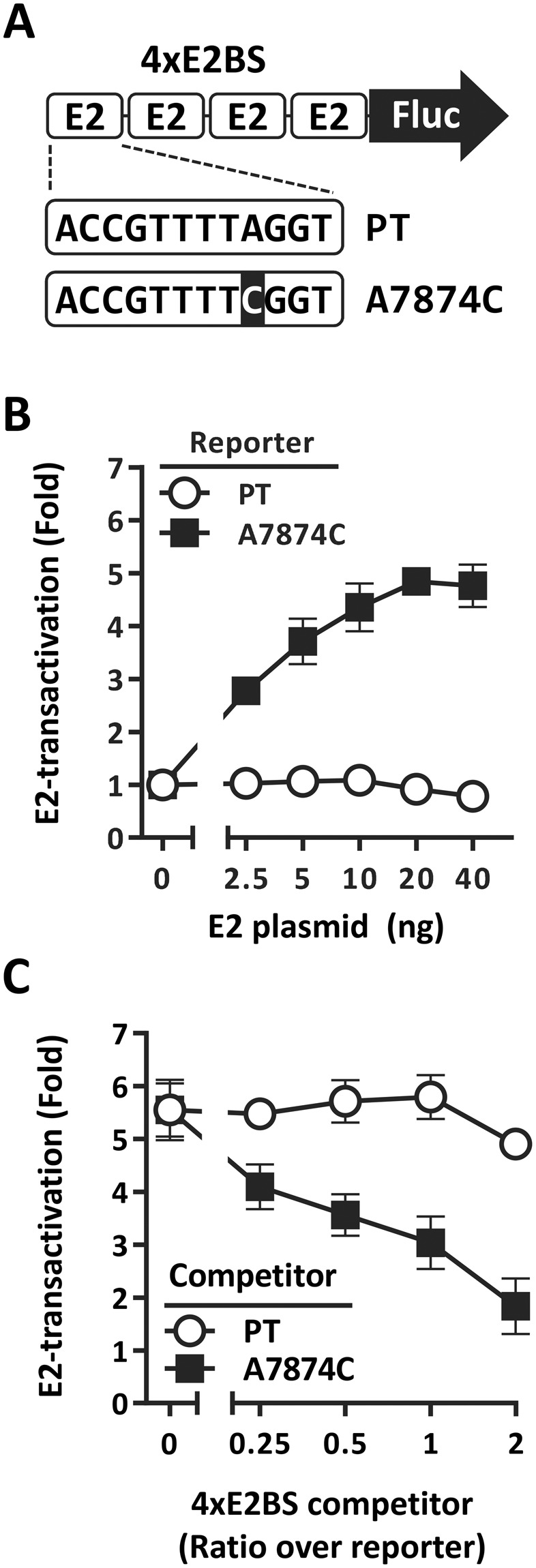
The A7874C variation enhances the activity of E2 at E2-binding site 2. (**A**) Schematic representation of the Fluc reporter plasmids containing four consecutive E2 binding sites (4 × E2BS), either of the prototype sequence (PT) or carrying the A7874C variation (highlighted in black). (**B**) Transactivation of the prototype or A7874C 4 × E2BS-Fluc reporter by E2. The ability of the HPV33 E2 protein to transactivate each reporter plasmid (160 ng) was measured in C33A cells using increasing amounts of E2-expression plasmid (0. 2.5, 5, 10, 20 and 40 ng). Each point represents the mean value obtained from 3 independent experiments performed in triplicates. Fold-transactivation values are calculated relative to the basal level of transcriptional activity obtained in the absence of E2, which was assigned a value of 1.0. Standard deviations are indicated by error bars. (**C**) Inhibition of E2-transactivation by 4 × E2BS competitor plasmids. The ability of the prototype (PT) and A7874C 4 × E2BS competitor plasmids to inhibit the transactivation of the A7874C-Fluc reporter plasmid (160 ng) by E2 (10 ng) was measured using increasing amounts of competitor plasmids (0, 80, 160 and 320 ng). Each point represents the mean value obtained from 2 independent experiments performed in triplicates. Fold-transactivation values and corresponding SDs are presented as described above.

The A7874C variation characterized above is located within a sequence that corresponds to E2 binding site 2 (E2BS2) of the HPV33 LCR (7866-ACCGTTTTAGGT-7877, A7874 underlined; Fig. 6A). To test if this variation affects the activity of E2 at this site, we constructed two firefly luciferase reporter genes under the control of four consecutive E2BS2 (4 × E2BS) either of the prototype sequence or containing the A7874C variation (Fig. 6A) and measured their level of transactivation by HPV33 E2 in C33A cervical carcinoma cells. As shown in Fig. 6B, the 4 × E2BS prototype reporter was transactivated very poorly by E2 (1.6-fold). In contrast, E2 activated the variant reporter 5.6-fold suggesting that A7874C enhances the affinity of E2 for this site. To verify this prediction, competition assays were performed with two plasmids carrying the four E2BS of the prototype and A7874C variant sequence, respectively, and lacking the Fluc ORF. Increasing amounts of each plasmid were tested for their ability to inhibit transactivation of the 4 × E2BS-Fluc A7874C reporter by E2. These experiments revealed that the plasmid containing the prototype 4 × E2BS was a poor competitor (Fig. 6C), thus confirming that it does not bind E2 efficiently. In contrast, the plasmid containing the variant A7874C 4 × E2BS reduced E2-transactivation of the reporter gene in a dose-dependent manner (Fig. 6C), with a 50% inhibition achieved at a ratio of competitor/reporter plasmid of 1:1. Taken together, these results indicate that the A7874C variation enhances the binding and activity of E2 at E2BS2.

## Discussion

Pioneering studies on HPV16 have validated the concept that nucleotide variations in the viral genome can alter the oncogenicity of variant viruses by affecting the activities of the viral oncoproteins and/or by tweaking their expression from the EP^21^. Although most variations in the LCR change the activity of the EP by 2- to 3-fold at the most, in reporter gene assays, these small differences were found to have a substantial impact on the replication and copy number of viral episomes as well as on their capacity to immortalize primary cells^10,11^. These findings highlighted the modulatory effects that LCR variations can have on the EP and how relatively modest changes in viral early gene expression can alter the biology of the viral genome.

**Figure 7:**
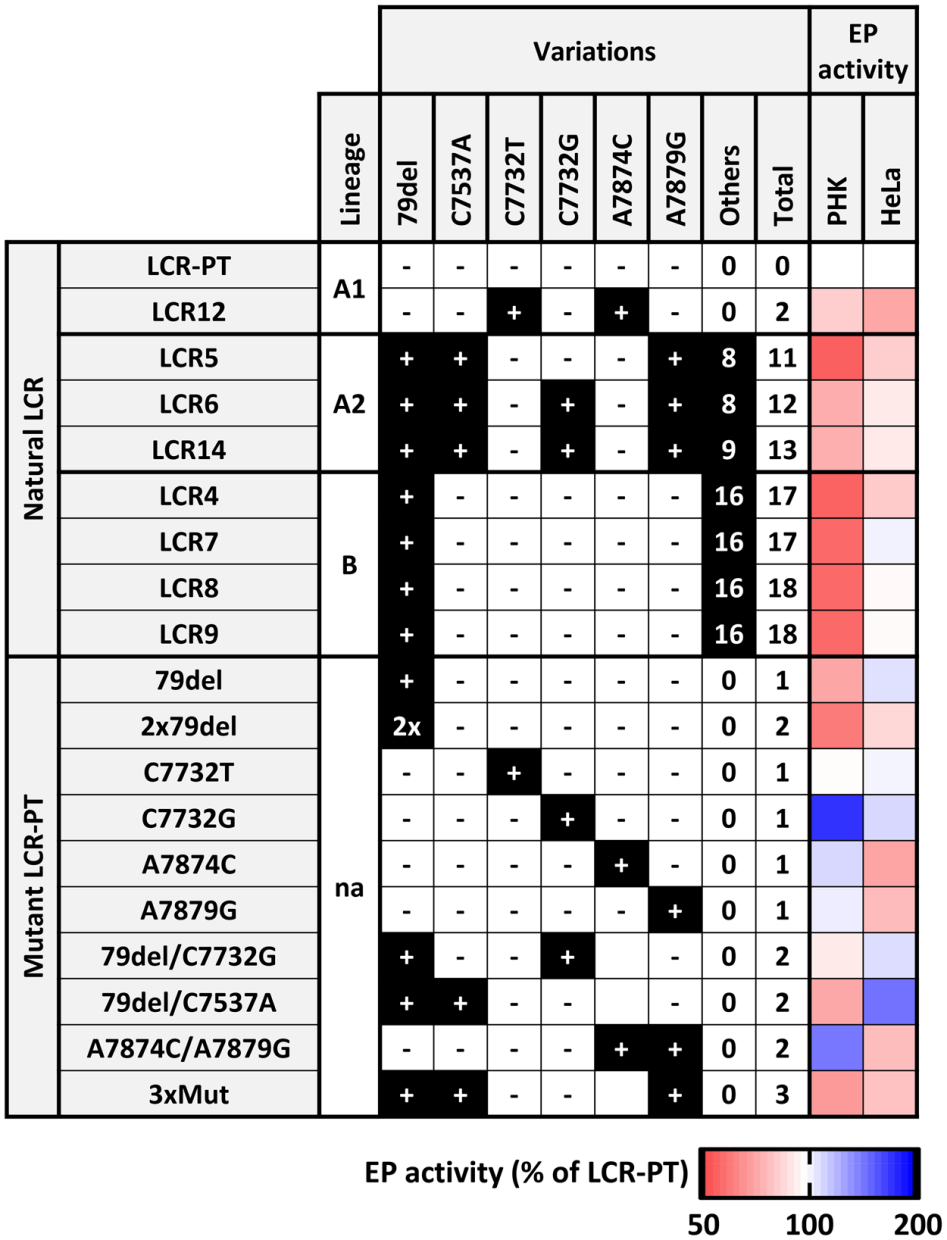
Heatmap summary of the EP activities of HPV33 variant and mutant LCRs. EP activities of the indicated LCRs are summarized in the form of a heatmap relative to the activity of the prototype (LCR-PT) which is assigned a reference value of 100% and colored in white in both PHK and HeLa cells. EP activity values lower and higher than 100% are colored with proportional intensities of blue and red, respectively, according to the legend shown at the bottom of the figure. Variations present and absent in each LCR are indicated by a plus sign on a black background (+) and by a minus sign (−), respectively. The 3xMut LCR carries the variations 79del, C7537A and A7879G. The number of additional variations (Others) and total number of variations in each LCR are indicated. The lineage of each LCR is indicated unless not applicable (na), as in the case of mutant LCR-PT created by site-directed mutagenesis.

The studies mentioned above also revealed that the activity of the EP measured in reporter-gene assays is a reasonable surrogate of its activity in the context of the whole genome^10,11^. This comforted us in the use of reporter-gene assays to measure the transcriptional activity of the HPV33 EP from the prototype and from eight variants representative of the worldwide diversity of the virus. PHK and HeLa cells were used in these experiments to assess the strength of the EP in the natural host (PHK) and its deregulation in cervical carcinoma cells (HeLa). Although our results revealed important differences between variant LCRs, our experimental approach and cell culture models are not without limitations and may therefore have missed some of the regulatory mechanisms that impinge on the EP during a normal infection or its progression to cancer. The transcriptional activities measured in this manuscript are summarized in Fig. 7, in the form of a heatmap where EP activities relative to the prototype (set at 100% in both cell types and colored in white) are indicated by different intensities of blue for the more active LCRs and of red for the less active ones. Two general observations can be made from this data. First, all variant LCRs displayed lower or similar EP activities compared to the prototype, in both PHK and HeLa cells. Second, many variant LCRs and mutant LCR-PT derivatives generated by site-directed mutagenesis showed different activities in PHK than in HeLa cells, thus emphasizing the deregulation of the EP in HeLa cells. This conclusion is also supported by the 3.5-fold higher activity of the EP in HeLa cells than in PHK (Fig. 1C).

Our characterization of HPV33 LCR variants was motivated by the previous associations of A1-sublineage variants with cervical intraepithelial neoplasia of grades 2–3 (CIN2/3)^22^ and cervical cancer^20^, and the results of our own studies, which revealed that the LCR variations C7732G and 79del are linked to HSIL and persistent infections, respectively^12,13^. Interestingly, the LCRs with the highest activities in PHK (white or lighter shades of red in Fig. 7) were the A1-sublineage LCR-PT and LCR12, and the two A2-variants LCR6 and LCR14 which contain the C7732G variation. Given the association of A1-viruses with CIN2/3 and cervical cancer^20,22^, and of C7732G with HSIL^13^, it is tempting to speculate that the stronger activity of these LCRs in PHK is reflective of their higher oncogenicity in epidemiological studies. This suggestion is further supported by our findings that the duplication of the 79-bp region in A1-variants, and the C7732G variation found in some A2-variants, are the two changes with the greatest stimulatory effect on the EP in PHK. Our results also indicate that C7732G contributes to the functional heterogeneity of A2-sublinage variants, with the two LCR variants containing this variation (LCR6 and LCR14) showing comparable activities as A1-variant LCRs, and the one lacking it (LCR5) behaving more like a B-lineage variant in PHK. Thus, we suggest that the presence or absence of C7732G should be taken into consideration in future epidemiological studies as it underlies the functional heterogeneity of A2-variants and, thus, may explain why this sublineage has not been associated with neoplasia.

The 79del deletion is present in the majority of A2- and B-lineage LCRs reported to date, but absent from A1-variants. Although it seems counterintuitive that a variation associated with persistent infections is found in the less oncogenic variants rather than the more oncogenic ones, a recent study showed that the median duration of infection by A2- and B-lineage viruses is indeed longer than for A1-variants, by approximately 6 months^23^. This study further showed that persistence does not account for the association of A1-viruses with CIN2/3, suggesting that these variants are intrinsically more carcinogenic^23^. Thus, while the stimulation of the EP by the 79-bp duplication could contribute to the oncogenicity of A1 variants, the lower transcriptional activity of A2- and B-variants caused by 79del may increase their persistence.

The transcriptional activities of our LCR variants were also measured in the HPV18-positive HeLa cell line, where the HPV33 prototype EP showed comparable activity as the HPV18 EP. In these cells, the activities of the variant EPs were more homogeneous and closer to that of the prototype, with the only exception being the 50% weaker activity of LCR12 (Fig. 2C) caused by the A7874C variation (Fig. 5B). Thus, while the HPV33 EP is upregulated in HeLa cells compared to PHK, the overall effect of variations is attenuated, clearly indicating a deregulation of the promoter. The weaker effect of C7732G in HeLa cells, and of the partial (79del) or complete deletion (2xdel) of the duplicated 79-bp region, was also apparent when these variations were introduced into the prototype LCR. In contrast, the 79del/C7537A and A7879G variations were found to have a substantial effect on the EP when introduced individually into LCR-PT, but to antagonize each other within LCR6. Thus, the comparable activities of the prototype and variants LCRs in HeLa cells can be explained by the fact that some variations like C7732G and 79del only have a minor effect and by the observation that the more active variations antagonize each other as seen for 79del/C7537A and A7879G in A2-sublineage LCRs.

Consistent with the downregulation of A2-variant LCRs by A7879G in HeLa cells, we also found that the A1-sublineage LCR12 is repressed by A7874C, which affects a nucleotide only 5-bp away from A7879. Interestingly, A7874C lies within E2-binding site 2 (E2BS2) of the LCR and was shown here to increase the activity and binding of E2 at this site *in vivo*. This effect of A7874C is easily explained by the fact that it improves the fit of E2BS2 to the high-affinity E2-binding consensus sequence ACCGNNNNCGGT (variant position underlined)^24^. Consistent with our finding, the converse C to A mutation in E2BS1 of the BPV1 genome (ACCACACCCGGT, mutated position underlined) was previously found to decrease the affinity of BPV1 E2 for this site *in vitro*^25^.

In conclusion, we have shown in this study that HPV33 variants exhibit radically different EP activities in PHK than in the HPV18-positive HeLa cell line. We have highlighted the stimulation of the EP in PHK by the 79-bp duplication unique to the oncogenic A1-variants and by the HSIL-associated variation C7732G, which contributes to the heterogeneity of the A2-sublineage. We also revealed the positive and negative regulation of the EP by the 79del/C7537A, A7874C and A7879G variations in HeLa cells and the increased E2-binding affinity that A7874C confers to E2BS2. Altogether, these findings provide a molecular framework to inform the results of previous and future epidemiological studies and pave the way for the identification of host transcription factors affected by these polymorphisms.

## Methods

### DNA sequence and phylogenetic analysis of HPV33 LCR variants

HPV33 LCRs were amplified from cervical samples and sequenced, each in two independent experiments, essentially as described before^12,13^. Cervical samples were obtained specifically for research purposes and processed as described previously^12,13^. All participants provided written informed consent. The Research and ethics committees of the Centre Hospitalier de l’Université de Montréal approved the study. All experiments were performed in accordance with relevant guidelines and regulations. All sequence variations are reported according to the numbering scheme of the HPV33 reference genome (GenBank accession M12732.1). All LCRs contained the A81C transversion found in other HPV33 isolates; a C at position 81 is considered the introduction of specific nucleotide changes into the prototype correct nucleotide although it is mistakenly replaced by an A in the GenBank reference sequence. LCR sequences were aligned using Clustal Omega^20,26,27^ and a phylogenetic tree constructed with EvolView^28^.

### Plasmid construction and mutagenesis

The LCR-PT-Fluc and LCR-PT-Rluc plasmids, which contain the HPV33 prototype LCR (termed LCR-PT; nt 7094–7909 and 1–108 of the HPV33 reference genome) in front of the Firefly luciferase (Fluc) and Renilla luciferase (Rluc) coding regions, respectively, were constructed by PCR amplification of the LCR from the reference genome with primers 33LCR-MluI-FWD (5′-GGGACGCGTACGCAAAAAGGTTAAAAAATAACACTTTGTG-3′, MluI site underlined) and 33LCR-NcoI-RV (5′-GGCGTCTTCCATGGAGTCGTGCAGTACCTTACTGC-3′, NcoI site underlined) and cloning of the resulting amplicon between the MluI and NcoI restriction sites of the promoterless pGL3-Basic (Promega) and pGL3-Rluc plasmids. pGL3-Rluc was generated by amplifying the Rluc gene from the pRL vector^29^ using primers Rluc-NcoI-FWD (5′-GGGCCATGGATGACCAGCAAGGTG-3′, NcoI site underlined) and Rluc-XbaI-RV (5′-CCGCAGTCTAGATTACTGCTCGTTC-3′, XbaI site underlined) and subcloning of this amplicon between the NcoI and XbaI sites of pGL3-Basic so as to replace the Fluc ORF by that of Rluc. HPV33 LCR variants were amplified and cloned into pGL3-Rluc as described above for LCR-PT. When indicated, mutations in the LCR were introduced with the QuikChange Site-Directed Mutagenesis kit (Stratagene). Plasmids encoding the prototype LCRs of HPV types 11, 16, 18 and 31 upstream of Rluc were constructed using a similar strategy to that for HPV33 LCR-PT. The sequences of these LCRs are identical to those available in the Papillomavirus Episteme (PaVE) database (http://pave.niaid.nih.gov/#home [2018]^30^). The plasmid for expression of HPV33 E2 tagged at its N-terminus with a triple-Flag (3 F) epitope was constructed by inserting a codon-optimized E2 ORF (synthesized commercially; GenScript) between the BamHI-EcoRI sites of pCMV-3Tag-1a (Stratagene). The 4 × E2BS competitor plasmid, which contains 4 copies of E2BS2(5′-CTCGAG*ACCGTTTTAGGT*CAT*ACCGTTTTAGGT*CAT*ACCGTTTTAGGT*CAT*ACCGTTTTAGGT*CATAGATCT-3′, E2BS2 in italics), was obtained by commercial gene synthesis (GenScript) and received cloned into the EcoRV site of pUC57. A similar 4 × E2BS plasmid containing the A7874C variation in each of the four E2 BS2 was also synthesized. Fluc-reporter plasmids driven from the prototype and A7874C variant 4 × E2BS sequences were constructed by excising the 4 × E2BS sequences from pUC57 with KpnI and HindIII and subcloning them into pGL2-Basic (Promega) upstream of Fluc. All constructs were verified by DNA sequencing.

### Cell culture and transfection

PHK of epithelial origin were purchased from Cell Applications Inc. and grown in EpiVita Adult Keratinocytes Growth Medium (cat. No. 141–500a, Cell Applications Inc.) as recommended by the manufacturer. The HeLa and C33A cell lines, which were authenticated by genetic analysis of 15 autosomal short tandem repeat loci and the gender identity locus amelogenin (by the service provider Genetica Cell Line Testing - a LabCorp brand), were grown in Dulbecco’s Modification of Eagle’s Medium (DMEM) supplemented with 10% fetal bovine serum (FBS), 50 I.U. of penicillin/ml, 50 µg of streptomycin/ml and 2 mM of l-glutamine (Wisent Bioproducts). PHK were transfected with the Cytofect-Epithelial Cell Transfection Kit (cat. No. TF102K, Cell Applications Inc.) while HeLa and C33A cells were transfected using the Lipofectamine 2000 reagent (cat. no. 11668-500, Life Technologies) according to the manufacturer’s recommendations.

### Luciferase-reporter gene assay

PHK and HeLa cells were plated in white flat-bottom 96-well plates (cat. no. 3917, Corning) at a density of 10 000 and 15 000 cells/well, respectively, 24 h prior to transfection. Cells were transfected with increasing amounts of each LCR-Rluc plasmid (25, 50, 100 and 200 ng) and a fixed amount of LCR-PT-Fluc (25 ng) as an internal control. The pGL3-Rluc plasmid (200 ng) was used as a negative control. Both Fluc and Rluc activities were measured with the Dual-Glo luciferase assay system and a GloMaxTM 96-well luminometer (Promega) 24 h post transfection. The Rluc/Fluc ratios determined at increasing amounts of each LCR-Rluc plasmid were used to calculate an area-under-the-curve (AUC) value that is reported as an overall activity of each LCR. The EP activity of each LCR-Rluc plasmid was tested in triplicates in at least 2 independent experiments in PHK, and in duplicates in at least 5 independent experiments in HeLa cells. Statistical significance was tested by ANOVA with Dunnett’s post-hoc analysis.

### E2 transactivation assay

C33A cells were plated in white flat-bottom 96-well plates (cat. no. 3917, Corning) at a density of 25 000 cells and co-transfected 24 h later with the prototype or A7874C 4 × E2BS reporter plasmid (160 ng), pRL control vector (0.5 ng), and increasing amounts of 3F-E2 expression plasmid (0, 2.5, 5, 10, 20, 40 ng). Dual-luciferase measurements were performed 24 h post transfection, as described above. Competition experiments were performed similarly, using the A7874C 4 × E2BS Fluc reporter plasmid (160 ng) and increasing amounts (0, 80, 160 and 320 ng) of competitor 4 × E2BS plasmid (PT or A7874C in pUC57). We chose to perform these experiments in C33A cells because their higher transfection efficiency makes them more suitable for quantitative studies than PHK and because, unlike HeLa cells, they do not contain an integrated HPV genome like HeLa cells that could be repressed by exogenous E2 and confound the analysis.

### Statistical analysis

Statistics, AUC calculations and the graphical representation of the data in the form of a heatmap were all performed using GraphPad Prism version 7.03.

## Data Availability

The datasets generated during and/or analyzed during the current study are available from the corresponding author on reasonable request.

## References

[1] Bernard HU (2010). Classification of papillomaviruses (PVs) based on 189 PV types and proposal of taxonomic amendments. Virology.

[2] Bosch FX (2008). Epidemiology and natural history of human papillomavirus infections and type-specific implications in cervical neoplasia. Vaccine.

[3] de Martel C, Plummer M, Vignat J, Franceschi S (2017). Worldwide burden of cancer attributable to HPV by site, country and HPV type. Int J Cancer.

[4] Hildesheim A, Wang SS (2002). Host and viral genetics and risk of cervical cancer: a review. Virus Res.

[5] Wang S. S., Hildesheim A. (2003). Chapter 5: Viral and Host Factors in Human Papillomavirus Persistence and Progression. JNCI Monographs.

[6] Ho L (1993). The genetic drift of human papillomavirus type 16 is a means of reconstructing prehistoric viral spread and the movement of ancient human populations. J Virol.

[7] Vande Pol SB, Klingelhutz AJ (2013). Papillomavirus E6 oncoproteins. Virology.

[8] Roman A, Munger K (2013). The papillomavirus E7 proteins. Virology.

[9] Kammer C, Warthorst U, Torrez-Martinez N, Wheeler CM, Pfister H (2000). Sequence analysis of the long control region of human papillomavirus type 16 variants and functional consequences for P97 promoter activity. The Journal of general virology.

[10] Hubert WG (2005). Variant upstream regulatory region sequences differentially regulate human papillomavirus type 16 DNA replication throughout the viral life cycle. J Virol.

[11] Lace MJ (2009). Upstream regulatory region alterations found in human papillomavirus type 16 (HPV-16) isolates from cervical carcinomas increase transcription, ori function, and HPV immortalization capacity in culture. J Virol.

[12] Gagnon S (2004). Viral polymorphism in human papillomavirus types 33 and 35 and persistent and transient infection in the genital tract of women. J Infect Dis.

[13] Khouadri S (2006). Human papillomavirus type 33 polymorphisms and high-grade squamous intraepithelial lesions of the uterine cervix. J Infect Dis.

[14] Schlecht NF (2001). Persistent human papillomavirus infection as a predictor of cervical intraepithelial neoplasia. JAMA: the journal of the American Medical Association.

[15] Ottinger M (2009). Cell-type specific transcriptional activities among different papillomavirus long control regions and their regulation by E2. Virology.

[16] Schenker A, Straub E, Iftner T, Stubenrauch F (2013). Cell-type-dependent activities of regulatory regions and E2 proteins derived from carcinogenic and non-carcinogenic human alphapapillomaviruses. The Journal of general virology.

[17] Bromberg-White JL, Meyers C (2003). Comparison of the basal and glucocorticoid-inducible activities of the upstream regulatory regions of HPV18 and HPV31 in multiple epithelial cell lines. Virology.

[18] Sun Z (2013). Genetic variations of E6 and long control region of human papillomavirus type 16 from patients with cervical lesion in Liaoning, China. BMC cancer.

[19] Lopez-Saavedra A (2009). Functional implication of sequence variation in the long control region and E2 gene among human papillomavirus type 18 variants. Archives of virology.

[20] Chen Z (2011). Evolution and taxonomic classification of human papillomavirus 16 (HPV16)-related variant genomes: HPV31, HPV33, HPV35, HPV52, HPV58 and HPV67. PLoS One.

[21] Burk RD, Harari A, Chen Z (2013). Human papillomavirus genome variants. Virology.

[22] Xi, L. F. *et al*. Lineages of oncogenic human papillomavirus types other than type 16 and 18 and risk for cervical intraepithelial neoplasia. *J Natl Cancer Inst* **106**10.1093/jnci/dju270 (2014). 10.1093/jnci/dju270 PMC4168311 25217779

[23] Xi LF (2016). Variant-specific persistence of infections with human papillomavirus Types 31, 33, 45, 56 and 58 and risk of cervical intraepithelial neoplasia. Int J Cancer.

[24] McBride AA (2013). The papillomavirus E2 proteins. Virology.

[25] Li R, Knight J, Bream G, Stenlund A, Botchan M (1989). Specific recognition nucleotides and their DNA context determine the affinity of E2 protein for 17 binding sites in the BPV-1 genome. Genes Dev.

[26] Raiol T (2009). Genetic variability and phylogeny of the high-risk HPV-31, -33, -35, -52, and -58 in central Brazil. Journal of medical virology.

[27] Vrtacnik Bokal E, Kocjan BJ, Poljak M, Bogovac Z, Jancar N (2010). Genomic variants of human papillomavirus genotypes 16, 18, and 33 in women with cervical cancer in Slovenia. The journal of obstetrics and gynaecology research.

[28] Zhang H, Gao S, Lercher MJ, Hu S, Chen WH (2012). EvolView, an online tool for visualizing, annotating and managing phylogenetic trees. Nucleic Acids Res.

[29] Fradet-Turcotte A, Morin G, Lehoux M, Bullock PA, Archambault J (2010). Development of quantitative and high-throughput assays of polyomavirus and papillomavirus DNA replication. Virology.

[30] Van Doorslaer K (2013). The Papillomavirus Episteme: a central resource for papillomavirus sequence data and analysis. Nucleic Acids Res.

